# Changes in substance use among adolescents before and during the COVID-19 pandemic in Guatemala

**DOI:** 10.3389/fpsyt.2024.1331962

**Published:** 2024-02-29

**Authors:** Jose Monzon, Joaquin Barnoya, Sophia Mus, Gustavo Davila, Desirée Vidaña-Pérez, James F. Thrasher

**Affiliations:** ^1^ Health Sciences School, Rafael Landívar University, Guatemala City, Guatemala; ^2^ Research Department, Cardiovascular Surgery Unit of Guatemala (UNICAR), Guatemala City, Guatemala; ^3^ Research Department, Integra Cancer Institute, Guatemala City, Guatemala; ^4^ Department of Health Promotion, Education, and Behavior, Arnold School of Public Health, University of South Carolina, Columbia, SC, United States; ^5^ Center for Population Health Research, National Institute of Public Health (Mexico), Cuernavaca, Mexico

**Keywords:** COVID-19, lockdown, smoking, adolescents, substance use

## Abstract

**Objectives:**

Due to the COVID-19 pandemic, on March 16^th^, schools had to be closed in Guatemala and went to online teaching. We sought to analyze the change in substance use among high school students in Guatemala associated with the lockdown.

**Methods:**

Data from two surveys (2019, n=2096, and 2020, n=1606) of a student cohort in private high schools in Guatemala City was used. Logistic models for past 30-day cigarette, e-cigarette, marijuana, and alcohol (including binge drinking) were used, regressing these on survey wave, while adjusting for sex, scholastic performance, high school year of student, parental education, substance use, and household member tobacco use.

**Results:**

Prevalence declined for smoking (10% to 3%, p<0.001), e-cigarette (31% to 14%, p<0.001), marijuana (4.3% to 1.9%, p<0.001), and alcohol use (47% to 38.5%, p<0.001), and binge drinking (24% to 13%, p<0.001). Adjusted models showed wave 2 associated with lower odds of using cigarettes (AOR=0.44, 95%CI=0.32-0.62), e-cigarettes (AOR=0.41, 95% CI=0.35-0.49, p<0.001), and binge drinking (AOR=0.73, 95%CI=0.59-0.89; p=0.002)

**Conclusion:**

Among Guatemalan adolescents, COVID-19 restrictions were associated with a significant decrease in smoking, e-cigarette use, and binge drinking.

## Introduction

Adolescence is a critical development stage of life that is accompanied by physical, cognitive, emotional, social, and behavioral changes (1). It is the peak time for substance use initiation, including smoking, alcohol, and other illicit drugs (2). Substance use might disrupt cognitive and emotional development and can also lead to substance use disorders, dependence, and poorer physical and mental health (1, 3) and has been reported as a leading risk factor for premature death globally (4). Substance use prevalence varies across the globe, for example, people from low-and middle-income countries (LMICs) as defined by the World Bank (5), were reported to have higher smoking prevalence than those in high-income countries (HIC) (6) due, in part, because of the rapid pace of globalization which facilitated the expansion of tobacco markets across borders (7, 8).

Electronic Nicotine Delivery Systems (ENDS) were first introduced in the market in 2007 (9, 10) and first documented in Guatemala in 2014 (11, 12) and now include Heated Tobacco Products (HTPs). Since then, consumption has increased, particularly in HIC. In the U.S., for example, e-cigarette use has been the most commonly used tobacco product among youths since 2014, and in 2020, 13.1% of middle and high school students were current users with 80% of them using flavored e-cigarettes (13). This increase is consistent with other countries as well (14–19) and in LMICs and upper-middle-income countries, like Guatemala (20). Even though Guatemala signed and ratified the Framework Convention on Tobacco Control (FCTC) (21), it lacks strong tobacco control strategies and ENDS remain unregulated. In 2014, 15.7% of adolescents were smoking compared to 5.6% using e-cigarettes (22). However, in 2019, we found a 27.7% e-cigarette and 8.7% cigarette use prevalence among adolescents (23). Therefore, it appears that consumption patterns shifted towards e-cigarettes in Guatemala City over time.

In March 2020, the World Health Organization (WHO) declared a pandemic the COVID-19 outbreak caused by the novel SARS-CoV-2 virus (24). To curb the pandemic countries (including Guatemala) implemented lockdowns (25), curfews (26), and school and workplace closures, among other non-pharmacological measures (27, 28). These measures also led to adolescent substance use (29, 30), including tobacco (31, 32), psychological distress (33–35), and economic impact (36, 37). Data on changes in adolescent substance use is for the most part from high-income countries (3, 13, 29, 38–41) and suggests a change and particularly a decline in smoking prevalence use during the pandemic (3, 38, 41–43). Possible explanations for this decrease include fewer interactions with peers, social gatherings, and access to retailers and advertising exposure. In addition, increased parental supervision and fear of COVID-19 are likely to play a role in decreasing smoking prevalence (38, 39, 42, 44).

In Guatemala, since the first COVID-19 case was detected in March of 2020, curfews, lockdowns, and building occupancy restrictions based on epidemiological indicators (45, 46) were implemented (47, 48). Schools were shut down on March 22^nd^, 2020, and turned to a fully virtual mode throughout that year until 2021. Regarding tobacco control, Guatemala has only a weakly enforced smoke-free environment law and consequently a frail tobacco control program (49). In addition, e-cigarettes are readily available and heavily advertised at the point of sale (50). Therefore, we sought to assess whether the COVID-19 school shutdown influenced adolescent cigarette, e-cigarette, marijuana, and alcohol (including binge drinking) use in a sample of high school students in Guatemala City.

## Methods

We analysed data from an ongoing, open cohort of students attending private schools in Guatemala City (51). Paper surveys were conducted in person from May to September 2019 (wave 1) and online from June to November 2020 (wave 2). Of the 2666 students, 1036 students were surveyed at both waves, and 1630 were surveyed once, due to maturation (out of and into high school) or absence during survey administration (n=3702 observations including those surveyed 2 times plus those surveyed only once). Passive parental consent was obtained by asking parents to respond to a consent letter only if they wished their kid to be removed from the study. After that, students provided active assent as described elsewhere (23, 52). Study protocols and data collection instruments were approved by the Institutional Review Board of Central America and Panama Nutrition Institute (INCAP).

The questionnaire focused on smoking and e-cigarette use and their risk factors, including alcohol and marijuana use. It was adapted from the Population Assessment of Tobacco and Health (PATH) study (53) and previously implemented in Mexico (54) and Guatemala (23). Current use was defined as using any product (cigarettes, e-cigarettes, marijuana, and alcohol) in the past 30 days. Participants were asked about their marijuana used as “during the past 12 months, have you smoked marijuana?” For the analysis, it was coded as a dichotomic variable (yes/no). We assessed binge drinking by asking “Have you ever had about 4 or more drinks on one occasion over the prior 30 days?”. They also reported on smoking and vaping among household members and their five closest friends dichotomized variables as follows: (1) At least one household member smokes; (2) at least one household member uses e-cigarettes; (3) at least one friend smokes; (4) at least one friend uses e-cigarettes. Sociodemographic variables included gender, grade (7^th^ to 11^th^), scholastic performance (from 1-100), and parental highest educational attainment (None, middle school and high school, some college and bachelor’s degree or more).

For the descriptive analysis, we compared sample characteristics across survey waves using omnibus chi-square statistics. We used logistic Generalized Estimating Equations (GEE) to estimate the influence of the COVID-19 lockdown over the use of cigarettes, e-cigarettes, marijuana, alcohol, and binge drinking. Models were adjusted for repeated measures and clustered within individuals using an exchangeable correlation structure. Models were adjusted for school performance, friends, and household member smoking and/or vaping, and sociodemographic characteristics. In total, we fitted 5 logistic GEE, one for each outcome assessed, all models were also adjusted for the use of the other substances that were not modeled as the outcome. All analyses were conducted in Stata version 17 (StataCorp LLC, College Station, Texas).

## Results

The response rate was 87% for wave 1 and 70% for wave 2. When comparing both waves (**Table 1**), there was a decrease in the reported prevalence of smoking (10% to 3%, p<0.001), e-cigarette (31% to 14%, p<0.001), marijuana (4.3% to 1.9%, p<0.001), alcohol use (47% to 38.5%, p<0.001), and binge drinking (24% to 13%, p<0.001). There was also a decrease in the friends and household members reported smoking (59% to 47% and 46% to 37%, respectively) and vaping (67% to 59%, and 24% to 18%, respectively) (**Table 1**).

**Table 1:** Demographic characteristics and substance use in Wave 1 and Wave 2 of adolescents’ cohort in Guatemala City.

	Wave 1 %	(n=2096)	Wave 2 %	(n=1606)	p-value
Sex					
Female	50.05%	(1,049)	53.74%	(863)	.213
Male	49.95%	(1,047)	46.26%	(743)	
Age					<.001
Less than 13	4.78%	100	1.59%	26	
13-15	44.29%	928	39.78%	638	
More than 15	50.94%	1068	58.63%	942	
Academic performance (average score on a 1-100 scale					
Less than 60 points	0.14%	(3)	0.06%	(1)	.055
60-69	2.00%	(42)	2.05%	(33)	
70-79	14.50%	(304)	13.95%	(224)	
80-89	48.47%	(1,016)	44.96%	(722)	
90-99	34.88%	(731)	38.98%	(626)	
At least one parent with high school education	87.74%	(1,839)	85.18%	(1,368)	.042
Substance use					
Current smokers	10.02%	(210)	2.99%	(48)	<.001
Current e-cigarette users	31.11%	(652)	14.13%	(227)	<.001
Current marijuana smokers	4.34%	(91)	1.93%	(31)	<.001
Alcohol consumers (at least once in previous month)	46.95%	(984)	38.48%	(618)	<.001
Binge drinkers (i.e. 4 or more drinks in a setting)	23.66%	(496)	13.20%	(212)	<.001
Advertising exposure					
Cigarette advertising	11.35%	(238)	4.67%	(75)	<.001
E-cigarette advertising	17.75%	(372)	8.97%	(144)	<.001
At least 1 household member smokes	45.47%	(953)	36.49%	(586)	<.001
At least 1 household member uses e-cigarettes	24.28%	(509)	18.43%	(296)	<.001
At least one friend smokes	58.87%	(1,234)	47.07%	(756)	<.001
At least one friend uses e-cigarettes	66.84%	(1401)	59.09%	(949)	<.001

**Table 2** shows the results of the adjusted logistic regression models showing the odds ratios of past 30-day substance use among adolescents. Wave 2 was associated with lower odds of reported smoking (AOR=0.44, 0.32-0.62; p=<.001), e-cigarette use (AOR=0.41, 95% CI=0.35-0.49; p=<.001), and binge drinking (AOR=0.73, 95% CI=0.60-0.89; p=.002). However, time-related differences in the use of marijuana (AOR 0.72, CI=0.47-1.1) and alcohol (AOR=0.91, CI=0.79-1.05) were no longer statistically significant after adjustment.

**Table 2:** Adjusted Odd Ratios of past 30-day substance among adolescents.

	Adjusted ORs (95%CI)	p-value
Electronic-cigarette use		
Male	1.41 (1.15-1.73)	<.001
School performance^a^	0.80 (0.70-0.91)	<.001
Parent education^b^	0.96 (0.72-1.27)	.76
Respondent High School Grade	1.23 (1.13-1.35)	<.001
Wave 2	0.41(0.34-0.49)	<.001
At least one friend uses e-cigarettes	5.62 (4.19-7.53)	<.001
At least one e-cigarette user in the household	2.72 (2.19-3.39)	<.001
E-cigarette ad exposure at least frequently^c^	1.24 (0.96-1.59)	.10
Current Smoker^d^	4.91 (3.44-6.99)	<.001
Alcohol Consumption^e^	4.57 (3.71.5.63)	<.001
Smoked marijuana in previous month	2.47 (1.55-3.94)	<.001
Cigarette Smoking		
Male	1.13 (0.82-1.55)	.46
School performance	0.77 (0.64-0.94)	.01
Parent education^b^	0.97 (0.60-1.57)	.91
Respondent High School Grade	1.17 (1.00-1.38)	.06
Wave2	0.47 (0.34-0.66)	<.001
At least one friend smokes	3.26 (2.18-4.87)	<.001
At least one smoker in the household	1.94 (1.42-2.65)	<.001
E-cigarette ad exposure at least frequently^c^	1.38 (0.89-2.15)	.15
Current e-cigarette user^d^	5.25 (3.68-7.50)	<.001
Alcohol consumption^e^	3.83 (2.41-6.08)	<.001
Smoked marijuana in previous month	4.59 (2.82-7.45)	<.001
Alcohol Consumption^d^		
Male	0.79 (0.67-0.93)	.01
School performance	0.92 (0.82-1.02)	.11
Parent education^b^	1.27 (1.00-1.62)	.05
Respondent High School Grade	1.64 (1.52-1.76)	<.001
wave2	0.92 (0.80-1.06)	.27
Current smoker^d^	4.23 (2.65-6.74)	<.001
Current e-cigarette user^h^	6.27 (5.14-7.65)	<.001
Smoked marijuana in previous month	3.36 (1.80-6.26)	<.001
Binge Drinking^g^		
Male	1.04 (0.84-1.29)	.74
School performance	0.91 (0.78-1.06)	.220
Parent education^b^	1.14 (0.81-1.62)	.448
Respondent High School Grade	1.83 (1.65-2.03)	<.001
Wave2	0.73 (0.60-0.89)	.002
Current smoker^d^	2.63 (1.81-3.80)	<.001
Current e-cigarette user^f^	9.40 (7.55-11.70)	<.001
Smoked marijuana in previous month	2.95 (1.66-5.25)	<.001
Smoking Marijuana		
Male	1.64 (1.08-2.49)	.021
School performance^a^	0.92 (0.69-1.21)	.535
Parent education^b^	0.65 (0.37-1.13)	.127
Respondent High School Grade	1.55 (1.22-1.98)	<.001
Wave 2	0.70 (0.45-1.09)	.118
At least one friend smokes cigarettes	4.39 (2.75-7.01)	<.001
At least one friend uses e-cigarettes	2.39 (1.47-3.88)	<.001
Alcohol consumption^c^	3.58 (1.88-6.82)	<.001

OR= Odds Ratio; CI=Confidence Interval

^a^Measured on a scale from 1-100

^b^At least one parent attended at least high school

^c^Responded at least frequently to the question: “in the last 30 days how frequently have you seen an e-cigarette ad?”

^d^Smoking at least once in the past 30 days

^e^Responded at least once to the question “In the last 30 days, how many days did you drink an alcoholic beverage?”

^f^Using an e-cigarette at least once in the past 30 days

^g^Had 4 or more drinks in one unique occasion in the past 30 days.

^h^E-cigarette use at least once in the past 30 days

## Discussion

To our knowledge, this is the first study assessing adolescent substance use before and during the COVID-19 restrictions in an upper-middle-income country in Latin America. We found that the reported smoking, vaping, marijuana and alcohol use, and binge drinking significantly decreased during COVID-19 restrictions. These declines remained significant in the adjusted models for all substances except alcohol and marijuana use. Although reports in other countries show mixed results (3, 41, 55–58), the US the National Survey on Drug Use and Health (NSDUH) did show a consistent decrease in adolescent substance use right before and after the COVID-19 restrictions (59). Similarly, in Ontario, Canada, data among 14-18-year-old adolescents before and after the emergency stay-at-home order in Ontario, yield a significant decrease (approximately 5%) in substance use after the shutdown (except for alcohol) (3). Even though these findings agree with what we found, these countries have significantly different tobacco control environments compared to Guatemala.

We also found that the reported friends’ and household members' substance use was significantly associated with teenagers' substance use, and they all significantly decreased in wave 2 (**Table 1**). This may be associated with a change in substance use during COVID-19 restrictions and is consistent with studies that suggest social networking as a significant means for initiation and continuation of substance use (60, 61) and particularly e-cigarettes. It also supports the idea that ENDS are generally obtained through peer networks and not from family members or direct purchasing as suggested elsewhere (62, 63).

The report of cigarette and e-cigarette use significantly declined in our sample, this makes sense during the lockdowns given that adolescent substance use is a highly social behaviour (57) and is consistent with another publication from Guatemala (64) a Canadian study in teens who provided information 3 weeks before and 3 weeks after the start of the COVID-19 lockdown (3).

Regarding alcohol use, binge drinking, and marijuana use, we also found a consistent decrease although in the adjusted models binge drinking decrease was not statistically significant. Other studies show mixed results, for instance, the Canadian study reported that fewer teens reported drinking, or using marijuana, however, alcohol use remained unchanged (3). Interestingly, among teens who did use substances, the mean number of days they used alcohol (0.76-0.96) and marijuana (0.94-1.1 days) were significantly higher after COVID-19 compared with before (3). Finally, another study among 12^th^ graders reported that adolescent marijuana and alcohol use had no significant changes during the pandemic despite decreases in the substances' perceived availability (55).

These mixed results underscore the importance of fully characterizing the effects of the pandemic on adolescent substance use patterns in the context of well-known risk factors for substance use such as drug availability, association with peers who use drugs, a lack of parental supervision, boredom, and coping with negative affect (44, 57) and be better prepared to address them.

Possible reasons that could explain our findings include that societal restrictions during the pandemic may have contributed to a decrease in the time youth spent with their peers, which ultimately has an impact on adolescent substance use though studies on this effect were done before the pandemic (65, 66). Another study suggested decreased commercial availability and access to vape devices which may be associated with decreased rates of vaping (39). Other studies however, attribute the increased amount of time spent within the household as a risk factor for familiar substance use exposure (67). These findings warrant future research on how significant policy changes affecting social behaviour (i.e. lockdowns) can affect substance use while also pointing to the fact that different substances may respond to its various determinants in a particular way and thus public policies will not function as a “one size fits all” for each substance.

## Limitations

This study should be interpreted considering some limitations. First, data from both waves is self-reported. However, this method has proved to be reliable to assess substance use in adolescents (68) and therefore internal validity should not be a source of bias. Also, we have reports that in 2019 and 2020, the Centers for Disease Control in the United States reported various outbreaks of Lung Injury Associated with the Use of E-cigarettes (EVALI), and thus we need to considerate that this could have also affected the e-cigarette use in adolescents in our sample, however, no reports of EVALI cases in Guatemala have been documented.

Furthermore, we changed survey implementation across waves, from in-person to online self-administration due to COVID-19 restrictions. It is yet unclear whether any resulting bias would have under- or over-estimated the changes we found. Finally, cigarettes and e-cigarettes utilize high-income status and exclusivity as part of its advertising and people with low-income status are less likely to afford these products and given that our sample was drawn from private schools in Guatemala City, our results might not be generalizable to public schools, lower-income status populations and rural Guatemala. Regardless, our findings provide evidence of the potential impact that the COVID-19 pandemic had in the substance use from our sample.

## Conclusion

Our results suggest that during the COVID-19 restrictions, substance use among Guatemalan adolescents declined, as well as smoking and e-cigarette use among peers and household members. However, declines in marijuana use and alcohol consumption appear independent of these influences and merit further investigation.

## Data availability statement

The raw data supporting the conclusions of this article will be made available by the authors, without undue reservation.

## Ethics statement

The studies involving humans were approved by Comite de Etica Instituto de Nutricion de Centroamerica y Panama. The studies were conducted in accordance with the local legislation and institutional requirements. Written informed consent for participation in this study was provided by the participants’ legal guardians/next of kin.

## Author contributions

JM: Conceptualization, Investigation, Methodology, Writing – original draft, Writing – review & editing. JB: Conceptualization, Funding acquisition, Supervision, Writing – review & editing. SM: Conceptualization, Investigation, Writing – review & editing. GD: Conceptualization, Writing – review & editing. DV: Data curation, Formal analysis, Writing – review & editing. JT: Conceptualization, Funding acquisition, Supervision, Writing – review & editing.
